# Do Fragile X Syndrome and Other Intellectual Disorders Converge at Aberrant Pre-mRNA Splicing?

**DOI:** 10.3389/fpsyt.2021.715346

**Published:** 2021-09-10

**Authors:** Sneha Shah, Joel D. Richter

**Affiliations:** Program in Molecular Medicine, University of Massachusetts Medical School, Worcester, MA, United States

**Keywords:** Fragile X syndrome, intellectual disability, autism, alternative splicing, gene regulation

## Abstract

Fragile X Syndrome is a neuro-developmental disorder caused by the silencing of the FMR1 gene, resulting in the loss of its protein product, FMRP. FMRP binds mRNA and represses general translation in the brain. Transcriptome analysis of the Fmr1-deficient mouse hippocampus reveals widespread dysregulation of alternative splicing of pre-mRNAs. Many of these aberrant splicing changes coincide with those found in post-mortem brain tissue from individuals with autism spectrum disorders (ASDs) as well as in mouse models of intellectual disability such as PTEN hamartoma syndrome (PHTS) and Rett Syndrome (RTT). These splicing changes could result from chromatin modifications (e.g., in FXS, RTT) and/or splicing factor alterations (e.g., PTEN, autism). Based on the identities of the RNAs that are mis-spliced in these disorders, it may be that they are at least partly responsible for some shared pathophysiological conditions. The convergence of splicing aberrations among these autism spectrum disorders might be crucial to understanding their underlying cognitive impairments.

## Introduction

Neurodevelopmental disorders (NDDs) including those that lie on the autism spectrum are diagnosed primarily based on behavioral presentations in early childhood. NDDs vary in severity of clinical presentations and most commonly occur as intellectual disability (ID) (1). Children with an autism spectrum disorder (ASD) often present with social and cognitive deficits, repetitive behaviors, language impairments, and intellectual disability that may range from mild to severe manifestations. The genetic heterogeneity and wide range of clinical presentations of ASD have hindered therapeutic advances. In 5–10% of ASD cases, the underlying causes are either single-gene mutations, chromosomal abnormalities, or environmental toxin exposure. Amongst the single-gene disorders, Fragile X Syndrome (FXS, caused by a triplet repeat expansion mutation in the FMR1 gene and subsequent loss of its protein product, FMRP) and the Rett syndrome (RTT, mutations in the MECP2 gene resulting in loss of MECP2 protein identified in 95% of the cases) are the most prevalent. Indeed 5% of ASD cases harbor the FXS mutation, and 50% of FXS patients are on the autism spectrum. About 50% of RTT patients are initially diagnosed with autism during the active regression period in Stage 2; however, upon progression to Stage 3; autistic features persist in only 19% of RTT patients (2). Understanding single gene intellectual disabilities (IDs) might aid in a better understanding of ASD, which currently has a prevalence of 1 in every 54 children aged 8 years in the US with co-diagnosis of ID in 33% of the children (3). Neurodevelopmental delays along with cognitive impairments are thought to stem from synaptic structure and function aberrations that are characteristic of ASDs and monogenic IDs such as FXS, RTT, tuberous sclerosis (TSC caused by mutations in the TSC1/TSC2 complex). Molecular analysis of patient-derived tissues and mouse models of the monogenic IDs has shown widespread changes at the epigenetic, transcriptional, and translational gene expression levels. The interplay between changes at multiple levels of gene regulation might be essential to the pathophysiology of a disorder. For example, in FXS, loss of FMRP protein resulting in increase in protein synthesis of FMRP target RNAs along with changes in multiple molecular pathways have been identified [reviewed in Ref. (4)]. Detailed molecular analysis of targets of translational de-repression in FXS led to the finding that a chromatin modifier, SETD2 (SET domain containing methyltransferase protein), is altered, resulting in downstream alterations in the chromatin landscape and genome-wide aberrant alternative splicing of mRNAs (5). Aberrant alternative splicing has previously been studied in complex NDDs such as ASD (6–8) and other psychiatric illnesses with a neurodevelopmental trajectory including Schizophrenia (8, 9), Bipolar Disorder (8, 9), Huntington's (10), and recently in monogenic IDs such as FXS (5), PTEN (11), and RTT (12–14) (Table 1).

**Table 1:** Evidence of differential alternative splicing in several neurodevelopmental disorders.

**Disorder**	**Origin tissue**	**Species**	**Mechanism**	**References**
Autism spectrum disorders	Frontal and temporal cerebral cortex	Homo sapiens	Regulation by splicing factors nSR100/Srrm4, RBFOX, and PTBP1 proteins	(6, 8, 15–17)
	Blood		–	(18)
Fragile X Syndrome	Hippocampal tissue slices	Mus musculus	Altered histone modifications at splice junctions of alternatively spliced exons (H3K36me3)	(5)
RTT	Hippocampus	Mus musculus	DNA modification 5hmC, and histone modifications (H3K4me3, H3K36me3)	(13, 19)
PTEN	Cortex	Mus musculus	Disruption of interactions with spliceosomal protein U2af2	(11)
Schizophrenia	Frontal and temporal cerebral cortex	Homo sapiens		(8, 9)
	Blood			(20)
Bipolar disorder	Frontal and temporal cerebral cortex	Homo sapiens		(8, 9)
	Blood			(20)
Huntington's disease	BA4 (Brodmann area 4) motor cortex	Homo sapiens	Regulated by splicing factor PTBP1	(21, 22)
		Drosophila melanogaster	Spliceosome proteins sequestered by mutant *Htt* mRNA.	(23)
Microcephaly	Fibroblast	Homo sapiens	Mutation in SNRPE gene results in failure to assemble the pre-mRNA processing complex U snRNPs.	(24)

*Studies using various human patient tissues as well as mouse models of causal mutations for IDs show widespread presence of aberrant pre-mRNA splicing*.

**Figure 1:**
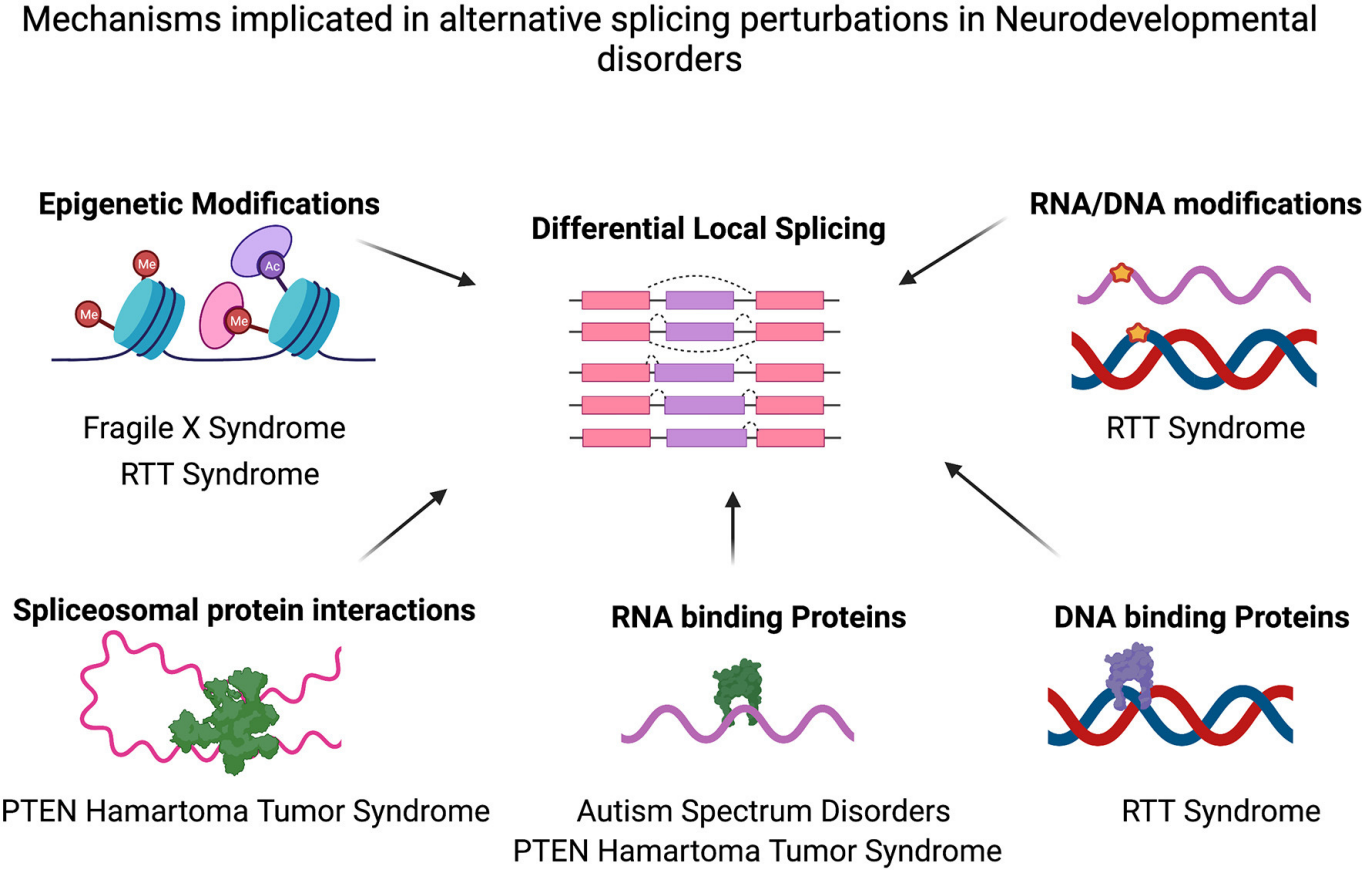
Mechanisms implicated in alternative splicing perturbations in various neurodevelopmental disorders. Differential alternative splicing results in various transcript isoforms generated from a single gene via splicing events (for example, Exon Skipping, Mutually exclusive exon usage, Alternative 3′ Splice site/5′ splice site usage or intron retention). Various molecular mechanisms may cause the genome-wide alternative splicing perturbations identified in several neurodevelopmental disorders such as epigenetic modifications (e.g., in FXS, RTT Syndrome) and/or splicing factor alterations (e.g., PTEN, autism) or as a result of DNA modifications and differential binding of proteins to DNA (RTT Syndrome). Figure created with Biorender.com. Also see Table 1.

Alternative pre-mRNA splicing generates multiple transcript isoforms and protein variants for a single gene by co-transcriptionally altering the exon composition of the mature mRNA (Figure 1). Alternatively spliced transcripts are widely prevalent in human cells and tissues, resulting in multiple transcript isoforms for 95% of the multiexon genes, generating a ~10-fold increase in the number of transcript isoforms per gene. With the advent of high-throughput transcriptomics, the alternative splicing landscape of cells and tissues can be readily investigated. Alternative splicing is detected in all metazoans and is closely correlated with organismal complexity (25). Furthermore, alternative splicing patterns are tissue- and cell-type-specific (10, 26), and in vertebrates, mainly contribute to the development and function of the central nervous system. Some neuronal genes such as Neurexins, n-Cadherins, and calcium-activated potassium channels can produce hundreds of mRNA isoforms resulting in a functionally diverse protein arsenal for efficient neuron functioning. Indeed, pathological consequences such as neurodegenerative, neurodevelopmental, and complex disorders including ASD occur when alternative splicing goes awry. Extensive studies using post-mortem patient brain tissue transcriptomics from ASD patients have shown pervasive mis-regulation of microexon (exon size of 3–27 nt) splicing (6, 7, 15, 27, 28).

Using genome-wide screens to identify RNA-binding proteins that might be key to the splicing dysregulation, splicing factors such as SRRM4/nSR100 were identified. A significant proportion of ASD cases also harbor *de-novo* genetic mutations resulting in cryptic splice sites or mutations in RNA binding proteins [e.g., *RBFOX1* (29)] that regulate alternative splicing. Deep neural networks such as SpliceAI can predict deleterious genomic mutations in complex disorders such as ASD (30). Studies from our lab on FXS and others' on RTT, PTEN have also identified alternative splicing aberrations in genes with essential neuronal functions, suggesting that improper transcript isoform ratios may be a key feature of several IDs. Given the recent increase in studies identifying alternative splicing defects, it might be useful to encourage parallel assessment of IDs to help focus on functionally relevant changes. Indeed, the biological pathways that local splicing defects might disrupt are similar amongst mouse models of multiple monogenic IDs and tissues derived from patients with complex disorders like ASD (for example, neuron development, synaptic vesicle function, cytoskeleton formation). They may thus underlie the shared pathophysiology of IDs.

A recent review has emphasized the ubiquitin system (UbS) as a potential convergent pathway among IDs such as Angelman Syndrome, FXS, PHTS, Loeys-Dietz Syndrome (LDS), and ASD (31). Furthermore, several epigenetic factors are misregulated in these disorders, which might trigger long-lasting effects on the epigenetic landscape in IDs. Translational regulation might be another point of convergence as loss of FMRP in FXS results in an increase in the synthesis of proteins, at least in some cases, by alleviating ribosomes from being stalled on specific mRNAs. Similar mechanisms of translational deregulation have recently been reported in Huntington's disease mouse models. Although many of these pathways are perturbed amongst different monogenic IDs, the most striking genome-wide alterations in complex IDs such as ASD are aberrant alternative pre-mRNA splicing. Understanding the potential regulatory factors and points of convergence among different IDs might help early identification, monitor progression, and perhaps lead to common therapeutic advances. Thus, we will highlight emerging research focus on alternative splicing in various IDs affecting neurodevelopment and brain function.

## Alternative Splicing During Neurodevelopment in IDS

Precise temporal regulation of alternative splicing of pre-mRNAs plays an essential role in every step of neurodevelopment, including but not restricted to cortical development (32), cerebellar development (33), neuron subtype specificity (34, 35), neuronal maturation (36), and axon formation (37). Tightly regulated ‘early switch' and ‘late switch' exons are encoded by genes in which exons are spliced out around birth or post-natally during development in neurons isolated from the mouse cortex during development, reflecting progressive stages of neuronal maturation (38). These switches may be orchestrated by an interplay of cell type-specific expression of various splicing factors (36, 38–41). Using integrative approaches to assess splicing data from various types of neurons at different stages of maturation, a ‘splicing code' has been proposed (38). Although the model currently uses only single exon changes, it does provide a foundation for future assessment of complex splicing patterns during neurodevelopment.

Another splicing program regulating mouse cerebellar cortex development and synapse maturation from P1 to P30 has been proposed to rely on the splicing factor SAM68 (33). Indeed, ablation of SAM68 resulted in impaired synaptic functions and long-lasting social interaction deficits in mouse models. Another splicing factor, nSR100/SRRM4, is crucial for regulating the alternative splicing program during neurodevelopment, especially microexon (3–27 nt) splicing (7). Microexons represent a class of exons that are enriched in protein-protein interaction domains that are associated with cell signaling and, due to their small size, have the highest tendency to be included in neuronal transcripts (6). An hypothesis for differential splicing of microexons (defined as <51 nt in this study) is that various genomic signatures such as the presence of intronic enhancers that dictate binding of splicing regulators including PTBP1 and RBFOX or alterations of the thymine content of the microexons may dictate their splicing patterns (16). Microexon mis-splicing occurs extensively in ASD, identified via large-scale transcriptomic studies from post-mortem cortex tissue from patients (6, 7, 11, 42). For example, the *CPEB4* (Cytoplasmic polyadenylation element binding proteins 4) gene regulates translation of mRNAs by altering their poly(A)-tails and plays an important role in embryonic development and synaptic plasticity. In ASD patients, a neuron specific microexon of *CPEB4* (Exon 4) which contains post-translational modification sites was found to be skipped to a greater extent resulting in changes in translation of downstream *CPEB4* target genes. Indeed, using a mouse model with deletion of *CPEB4*- Exon 4 a potential functional role for the microexon skipping was assayed. Mutant mice displayed changes in polyadenylated transcriptome corresponding to changes in high-risk expression ASD genes (e.g., *PTEN, RBFOX1, AUTS2*) and mimicked ASD phenotypes such as deficits in spine density, reduced number of excitatory synapses along with typical ASD behaviors such as stereotypic running and diminished social interaction (28). However, an important caveat to this study is that the physiological change in the exon 4 of *CPEB4* in ASD patient brain tissue was a modest exon inclusion difference (percent spliced in (PSI) change, ASD vs. control) = −7.6%. Similar to ASD, the inclusion level difference of exons in various IDs for most genes is modest, and hence their impact on pathophysiology needs further investigation.

Alternative splicing programs are also constantly altered during aging (43–45), and their disruption can result in the early onset of neurodegeneration (43, 44, 46). Thus, evaluating age-specific disruptions in alternative splicing patterns could help assess developmental delays in IDs. For example, assessing the splicing patterns in PTEN mouse models at P14 (post-natal day 14) and P40 (post-natal day 40) time points highlighted age-specific mis-spliced isoforms that might disrupt distinct signaling pathways, partly due to age-specific changes in expression of splicing factors (11). Studying temporal patterns of alternative splicing aberrations during neurodevelopment in IDs may be crucial to assess developmental delays and find critical periods to correct splicing deficits.

## Effect of Alternative Splicing on Brain Function in ID

Neuronal stimulation causes differential exon selection in mRNAs resulting in functionally diverse mRNA isoforms (47). Multiexon ion channel encoding mRNAs are among the most extensively spliced mRNAs in neuronal cells (48–54). Neuronal depolarization with KCl results in widespread alternative splicing changes in neuronal cultures *in-vitro*, affecting selective permeability of ion channels (33, 55, 56). Neuronal activity-dependent splicing changes may affect long-term synaptic plasticity. One example is activity-dependent splicing of RNAs encoding neurexins', a class of crucial trans-synaptic cell adhesion molecules that govern synapse assembly, transmission, and identity. Alternative splicing and alternative promoter usage result in thousands of neurexin (*Nrxn*) isoforms with potentially diverse functions (57). Membrane depolarization-induced shift in *Nrxn1* splice isoform choice via calcium/calmodulin-dependent kinase IV signaling altered trans-synaptic signaling regulated by SAM68 (a splicing factor) (56). Studies on alternative splicing in response to membrane depolarization in cerebellar neurons also identified SAM68 as a critical regulator of the splicing pattern (33). Neuronal activity-dependent alternative splicing is also highly dysregulated in ASD, specifically microexon (3–27 nt) splicing (15, 58). A mouse model mimicking microexon skipping in ASD for the translation initiation factor, eIF4G (Eukaryotic translation initiation factor 4 G), demonstrated activity-dependent exclusion of a microexon resulted in increased levels of synaptic proteins involved in mediating neuronal activity and synaptic plasticity (59).

Another neurodevelopmental disorder that shows prevalent alternative splicing changes that manifests with learning disabilities and autism is the Rett Syndrome (RTT) (12). RTT mainly occurs due to loss of the MeCP2 protein, which is studied using MeCP2 deficient mouse models. Widespread changes in alternative splicing were identified in MeCP2 null mice, which were exacerbated by membrane depolarization (14). Furthermore, MeCP2 null mice were more susceptible to seizures post-neuronal stimulation, which may be due to the altered splicing program. MeCP2 mediated alternative splicing may also regulate spatial learning-induced memory consolidation in hippocampal tissue (13). However, the contribution of alternative splicing defects in the MeCP2 null phenotype is still unclear (60). Highly congruent to ASD are the alternative splicing changes recently identified in the hippocampus from the Fragile X Syndrome mouse model [*Fmr1* knockout (KO)] (5). Synaptic vesicle localization and function were amongst the top enriched categories of differentially spliced genes. This could imply that, the synaptic plasticity deficits observed in *Fmr1* KO hippocampus tissue could be mediated by altered isoform ratios of key synaptic function genes.

## Mechanisms of Alternative Differential Splicing in ID

Alternative splicing regulation is a highly variable process that depends on complex interactions between cis-acting splicing signals, splicing factor recruitment, RNA motifs, combinatorial action of RNA binding proteins, epigenetic marks on the chromatin, speed of polymerase movement, and other molecular features. The mechanisms contributing to alternative splicing in the mammalian nervous system, which have been reviewed in Ref. (27), may explain the aberrant pre-mRNA splicing programs in IDs (Figure 1). Amongst the alternative splicing patterns examined in IDs, the most detailed have been in ASD. Studies from idiopathic ASD patient post-mortem brain tissue identified a reduction in nSR100/SRRM4 proteins in a significant proportion of tissue samples (6, 15). Mouse models mimicking haploinsufficiency of nSR100/SRRM4 (splicing regulators) also reproduced the misregulated splicing patterns seen in ASD patients and displayed deficits in social behavior (15). A study investigating alternative splicing defects in blood samples from autistic boys showed a set of 53 mis-spliced RNAs (18), several of which encode proteins that regulate differential alternative splicing. Altered expression of other splicing regulating factors, RBFOX1, RBFOX3, and PTBP1 have also been implicated in ASD (8, 16, 40, 41). Interestingly, mRNAs bound by RNA binding proteins, RBFOX1 and FMRP were differentially spliced in patient tissues from both ASD and Schizophrenia (8), suggesting that RNAs that show common splicing defects amongst these disorders may be targeted by similar processes.

Similar to ASD, a decrease in expression of splicing factor SRRM4 has also been identified in a PTEN mouse model along with decreased expression of other proteins such as NOVA2 and RBFOX1. Another hypothesis explaining the local splicing defects in PTEN, is that mutations in the *PTEN* gene might disrupt interactions of Pten with the spliceosomal protein U2AF2, thereby altering the local splicing program.

Alternative splicing of mRNAs can also be regulated by epigenetics, including histone modifications, DNA methylation, long non-coding RNAs posing as splicing factors, and potentially RNA modifications such as N6-methyladenosine (m6A), and DNA modifications such as 5-hydroxymethylcytosine (5hmC). In Rett syndrome, MeCP2-dependent spliced exons show differential epigenetic signatures based on their tendency of inclusion or exclusion. Excluded exons display the DNA modification 5hmC, and the presence of the histone modification H3K4me3 (histone H3 lysine 4 trimethylation) H3K4me3. Exons differentially included in the mature mRNA demonstrate enrichment of H3K36me3 (histone H3 lysine 36 trimethylation) marks in Mecp2-knockdown neurons (19). Similarly, epigenetic marks may contribute to the alternative splicing defects observed in brain tissues from the FXS mouse model. In FMRP deficient hippocampus tissues, one of the mRNAs released from translational repression is *SETD2*, resulting in increase of SETD2 (a lysine methyltransferase) protein levels. Increased SETD2 alters the H3k36me3 marks on the chromatin, which correlated with alternative splicing defects in mRNAs critical for proper synaptic function (5).

## Discussion

The functional significance of alternative splicing deficits in neurodevelopmental disorders is becoming increasingly clear owing to the ease of transcriptomic analysis and the use of mouse models mimicking exon mis-splicing events. An in-depth analysis of consolidated transcriptomic data from a large cohort of patients diagnosed with ASD, schizophrenia and bipolar disorder have provided significant insights enriching our understanding of the pathophysiology of psychiatric disorders (8). A key finding of this study was that rather than gene expression changes, isoform level aberrations were the most abundant, and demonstrated important functional gene category enrichments and disease specificity. Notably, the majority of the local splicing changes identified were restricted to each disorder, with only two genes that showed a splicing change in all three disorders. Amongst the pre-mRNA splicing aberrations identified in monogenic IDs, there is a significant overlap with those found in ASD patients, however majority of the splicing changes found are disorder specific. The process of differential alternative splicing resulting in dysregulated mRNA isoform expression maybe conserved amongst IDs, however, the exact genes may differ based on the different mechanisms that lead to the splicing deficits (Table 1; Figure 1). Thus, alternative splicing changes could potentially provide a molecular fingerprint to identify and differentiate among NDD pathologies. Growing numbers of studies have also identified differential pre-mRNA splicing in blood samples from patients (18, 20), suggesting the possibility of a potential biomarker for IDs and various NDDs. Given, the recent finding of differential splicing programs predominantly present in various IDs, a coordinated effort to generate and study large scale transcriptomics datasets will thus be beneficial. However, identifying all functional splicing changes in a given transcriptomics dataset is still a herculean task that relies primarily on identifying the protein domain encoded by the mis-spliced region. Considerations toward the extent of local splicing changes (ΔPSI, percent spliced-in) are imperative since the proportion of splicing change in isoforms, and the frequency of isoform expression, which often range from 10 to 40% in IDs, may not be enough to result in a functional outcome (protein expression or RNA localization). Whether a substantial ΔPSI change in one gene can result in a phenotype or smaller ΔPSI in multiple genes in the same pathway might contribute to disease manifestation would be helpful to understand. For example, although the ΔPSI of the differentially spliced mRNAs in the *Fmr1* KO mouse tissues were between 10 and 30%, most of the mRNAs were involved in synaptic vesicle recycling function in the hippocampus. Thus, the synaptic plasticity deficit in this tissue may in part be due to small splicing changes in many mRNAs in the same pathway. Furthermore, splicing analysis results may be subject to the choice of bioinformatic programs used to assess splicing changes, depth of sequencing, ΔPSI threshold, read length and type of sequencing, reference genome version, and sample characteristics such as age, tissue, and cell type studied. Consolidating alternative splicing patterns from different studies requires careful assessment of all factors that may contribute to a bias in data generation. Identification of similar alternative splicing changes in genes belonging to specific biological pathways such as synaptic function or brain development in different monogenic and complex neurological disorders, suggests that similar upstream regulatory pathways may be perturbed.

Dysregulated pre-mRNA splicing is a rapidly developing research focus in understanding pathophysiology of neurodevelopmental disorders. Several questions arise based on current research and answering them is vital to our understanding of how neurodevelopmental disorders manifest and show such phenotypic diversity in patients. For example, is aberrant splicing a cause or consequence of changes in the epigenetic landscape, splicing factor levels, or RNA binding proteins? Can restoring alternative splicing defects result in reversing the course of the disorder? If not, can the alternative splicing changes be used as biomarkers to inform therapeutic strategies and disease progression? Can the alternative splicing perturbations explain commonalities and differences between IDs and therefore predict the severity of the disorder? The genome-wide perturbations of alternative pre-mRNA splicing is a recent finding, and it is of particular importance to specifically study isoform level gene regulation to fully understand the molecular mechanisms of neurodevelopmental disorders.

## Data Availability Statement

The original contributions presented in the study are included in the article/supplementary material, further inquiries can be directed to the corresponding author/s.

## Author Contributions

The manuscript is written by SS. JR reviewed and provided substantial inputs to the manuscript.

## Funding

This research was supported by NIH Grants GM46779 and GM135087. SS was supported by a FRAXA Foundation postdoctoral fellowship.

## Conflict of Interest

The authors declare that the research was conducted in the absence of any commercial or financial relationships that could be construed as a potential conflict of interest.

## Publisher's Note

All claims expressed in this article are solely those of the authors and do not necessarily represent those of their affiliated organizations, or those of the publisher, the editors and the reviewers. Any product that may be evaluated in this article, or claim that may be made by its manufacturer, is not guaranteed or endorsed by the publisher.
